# The association between age at menarche and depression: a cross-sectional analysis of the TABARI cohort at enrollment phase

**DOI:** 10.1186/s12888-025-06667-w

**Published:** 2025-03-25

**Authors:** Mahmood Moosazadeh, Seyed Hamzeh Hosseini, Monirolsadate Hosseini Tabaghdehi, Masoomeh Shafiei, Erfan Ghadirzadeh

**Affiliations:** 1 Gastrointestinal Cancer Research Center, Non- Communicable Diseases Institute, Mazandaran University of Medical Sciences, Sari, Iran; 2 Department of Psychiatry, Psychosomatic Research Center, Sari Imam Khomeini Hospital, Faculty of Medicine, Mazandaran University of Medical Sciences, Sari, Iran; 3 Department of Midwifery, Health Reproductive Research Center, Sari Branch, Islamic Azad University, Sari, Iran; 4 Department of Obstetrics and Gynecology, Shiraz University of Medical Sciences, Shiraz, Iran; 5 Gastrointestinal Cancer Research Center, Non-Communicable Diseases Institute, Mazandaran University of Medical Sciences, P.O.BOX: 4816117949, Sari, Iran

**Keywords:** Depression, Age at menarche, Tabari cohort study

## Abstract

**Background:**

Puberty, particularly menarche, involves hormonal changes that may influence depressive symptoms. However, research on the association between age at menarche (AAM) and depression yields contradictory results, possibly due to sample differences and differences in socioeconomic status, parenting style, and cultural factors within each studied population. Thus, this study aimed to investigate the association between AAM and depression in a large cohort of the Northern Iranian population.

**Methods:**

This cross-sectional study comprised 6103 female adults aged between 35 and 70 years from the Tabari cohort study. The association between depression and three different AAM subgroups (≤ 11 as early menarche, 12–13 as normal menarche, and ≥ 14 as late menarche) was compared using logestic regression models after adjusted sociodemographic factors.

**Results:**

The crude model showed that females with early AAM and normal AAM had higher odds of depression (OR: 1.27, 95% CI: 0.96–1.69, *P* = 0.09, and OR: 1.21, 95% CI: 1.03–1.43, *P* = 0.024, respectively) compared to the late AAM group (P for trend = 0.042). However, in the fully adjusted model, there were no significant associations (OR: 0.97, 95% CI: 0.73–1.29, *P* = 0.827 for early versus late AAM, and OR: 0.98, 95% CI: 0.82–1.17, *P* = 0.830 for normative versus late AAM).

**Conclusion:**

Our results indicated that, while no significant relationship was observed between different AAM subgroups and depression in the multivariable model, there was a notable trend suggesting an improvement in depression with later AAM.

**Supplementary Information:**

The online version contains supplementary material available at 10.1186/s12888-025-06667-w.

## Background

Depression is a mental health disorder that can affect an individual’s overall well-being and result in irritability, fatigue, impaired judgment, social isolation and elevated risk of self-harm in the individual [1]. It has been reported that on average, 34% of adults have experienced depressive symptoms worldwide with the highest prevalence in female adults residing in Middle East, Asia, and Africa [2]. Recent studies in female Iranian population have demonstrated an average of 49% prevalence in depressive symptoms with significant differences regarding, socioeconomic status, parenting style, and education [3, 4].

Previous studies have searched for potential causes that makes females more vulnerable to depression including social, cultural, and biologic factors and hormonal variations [5, 6]. Puberty is a natural process of sexual maturation and involves a series of biological and psychological alterations [7]. Menarche is one of the significant milestones in the process of puberty in females which is associated with increases in sexual steroid levels [8]. Recent studies have shown that changes in sex steroids including estrogen in females could contribute to the development of depressive symptoms [9, 10]. Additionally, alterations in these sexual hormones may change the natural course of puberty and maturation including the timing of menarche [11, 12]. Thus, many studies hypothesized that there may be an association between age at menarche (AAM) and depression in females.

AAM might influence depression in adulthood due to both biological and psychosocial factors. Biologically, puberty is accompanied by significant hormonal changes, particularly in sex steroids such as estrogen and progesterone, which influence brain development and emotional regulation. Early menarche may result in an extended exposure to these hormonal fluctuations over the lifespan, potentially increasing susceptibility to mood disorders [13, 14].

Some studies suggested that late menarche is associated with depression [15], whereas others suggested otherwise and found early menarche to be a risk factor for depression [16]. For instance, Shen et al. [16] reported that females with early AAM have 1.25 times higher odds of developing depression compared to those with normal AAM, with no significant difference observed between normal and late AAM. In contrast, Herva et al. [15] found that females with late menarche have 1.7 times higher odds of depression compared to those with normal AAM, while no significant difference was observed between early and normal AAM. Additionally, there were also studies which states no associations between AAM and depression [17]. These contradictory results may have been due to differences in sample size, ethnicity, and cultural behaviors of the population. An overview of the controversies in the results of the previous studies has been demonstrated in Supplementary Table 1.

Most of the previous literature in this field were conducted in American, European, or Chinese population. Thus, due to differences in socioeconomic status, parenting style, and cultural factors, we aimed to investigate the association between AAM and depression in a large cohort registry of Northern Iran population.

## Methods

### Population and study design

This cross-sectional investigation utilized enrollment phase data from the TABARI cohort study (TCS), a subset of the larger Iranian national cohort known as the “Prospective Epidemiological Research Studies in Iran (PERSIAN).” The TABARI enrollment phase comprised 10,255 individuals (4149 males and 6106 females) aged 35 to 70, residing in urban and mountainous areas of Sari, the capital city of Mazandaran located in northern Iran, along the foothills of the Alborz mountain range and the Caspian Sea shores, during the period from 2015 to 2017. Employing a census-based sampling approach, requisite data were obtained through the TCS registration system. A standardized questionnaire, detailed in methodology articles and cohort profiles [18–20], was utilized in demographic data collection. Trained interviewers, who had participated in national and provincial workshops, administered the questionnaire either in-person or via standardized web-based (online) platforms, adhering to the PERSIAN cohort protocol. Additional information on the methodologies employed in the PERSIAN and TCS can be found in the cohort profile and methodology articles [18–20]. The entire female population of the TCS was included in this study, with exclusion of individuals with missing data and participants suffering from kidney failure or cancer.

### Measurements

In the current investigation, demographic data including age, residential area (urban/mountainous), socioeconomic status (lowest to highest), marital status, education level, occupation, physical activity (PA) level, and anthropometric assessments were extracted from the TABARI cohort data repository. Anthropometric indices such as height, weight, and body mass index (BMI) were assessed using standardized tools. Height measurements were obtained using the SECA 226 stadiometer (SECA, Hamburg, Germany), while weight measurements were conducted with the SECA 755 analogue standing scale (SECA, Hamburg, Germany).

AAM alongside other obstetric variables including use of oral contraceptives (OCP), age at first pregnancy or age at primigravida (APG), number of pregnancies (Gravida), and history of hysterectomy were documented in the TCS database. For this study, participants were categorized into three groups based on their AAM: early menarche (at age 11 or younger), menarche at age 12–13, or menarche at age ≥ 14.

### Depression assessment

Participants were identified as experiencing depression through a combination of self-reported data and medical records. During the enrollment phase of the TCS, participants were required to provide comprehensive documentation of their health documents and current medications. Those classified as depressed included individuals diagnosed with depression by a psychiatrist, those with a documented history of depression, and those currently taking antidepressant medications. It is noteworthy that in epidemiological research employing extensive sample sizes, reliance on documented self-reporting does not present substantial limitations. It is also important to note that self-reported depression data in the TCS has undergone validation. In this validation process, the sensitivity, specificity, and accuracy of self-reported depression were determined to be 95.6%, 53.7%, and 78.1%, respectively, for women [21].

### Statistics

The data analysis was conducted using SPSS software version 26 (IBM SPSS Corp, USA). Variables were described using frequencies and percentages. Participants AAM was categorized into three levels: less than 11 years (early menarche), 12–13 years (normal menarche age), and 14 years and above (late menarche age). Group comparisons were made using the Chi-square test. The crude odds ratio (OR) of depression based on each investigated variable was calculated using logistic regression. The OR of depression by AAM groups, accompanied by P for trend, was adjusted for potential confounders such as age group, residential area, socioeconomic variables, education level, occupation, marital status, menopausal status, BMI, physical activity, APG, number of pregnancies, OCP use, and hysterectomy using logistic regression. Additionally, factors associated with depression were presented using a multivariable logistic regression model.

### Ethics

This study was conducted without commercial input or involvement in the design, implementation, analysis, or reporting. This study was approved by the Research Ethics Committees of Mazandaran University of Medical Sciences (Ethics Approval Code: 2524). Written informed consent was obtained from all participants before entering the study.

## Results

In this study, a total of 6103 women from the TCS were included in the analysis. Among them, 683 individuals (11.2%) reported a history of depression based on prior diagnosis in which, 620 individuals (90.8% of the 683) were under treatment. The mean age at diagnosis for depression was 39.38 ± 10.69 years. Additionally, 528 individuals (8.7%) had early AAM (≤ 11 years old), 2379 individuals (39%) had normal AAM (12–13 years), and 3196 individuals (52.4%) had late AAM (≥ 14 years old).

**Fig. 1:**
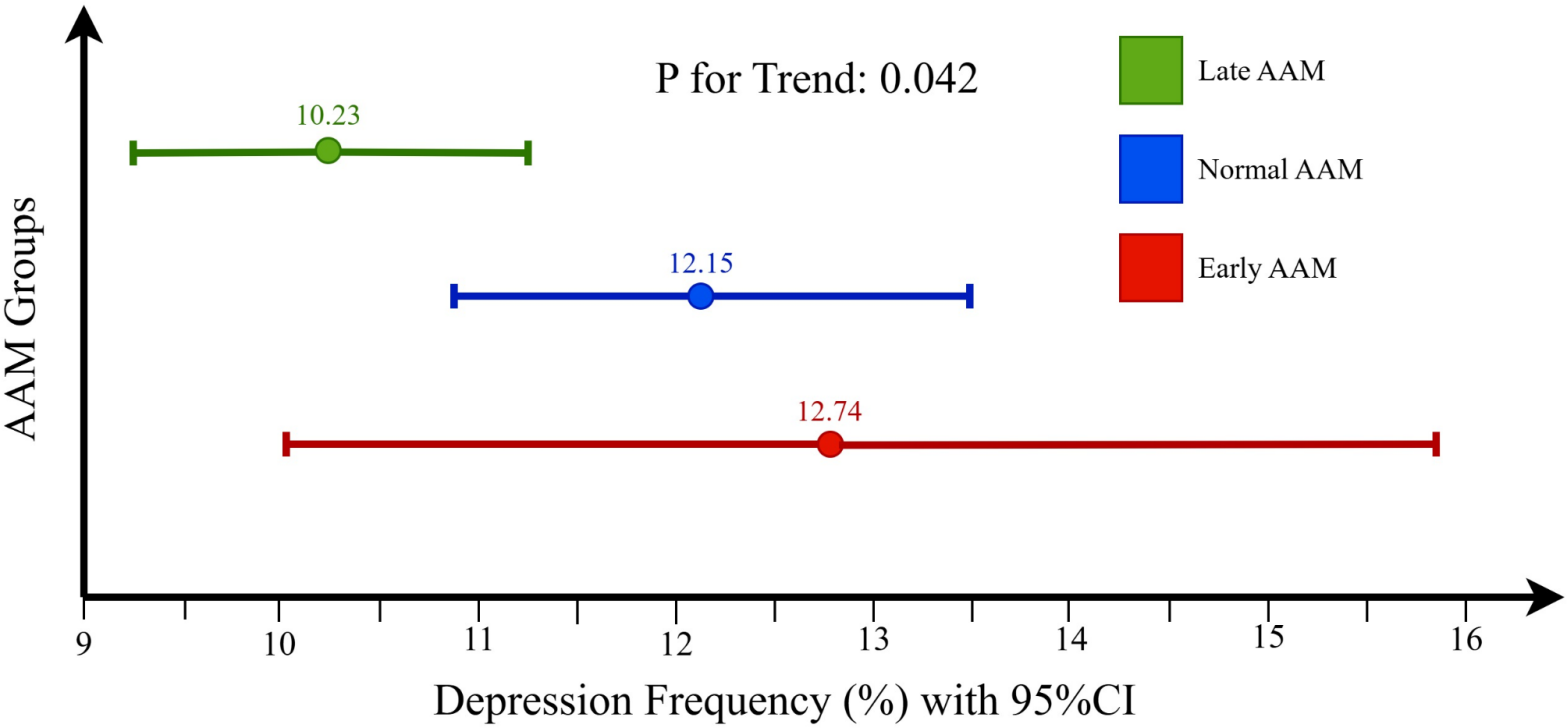
Trends in depression frequency based on AAM groups

The prevalence of depression was 12.74% (95%CI: 10.01–15.89) (67 individuals out of 528) among females with early AAM, 12.15% (95%CI: 10.86–13.53) (289 individuals out of 2379) among females with normal AAM, and 10.23% (95%CI: 9.20-11.33) (327 individuals out of 3196) among those with late AAM (P for trend = 0.042) (Fig. 1). Univariate logistic regression showed that the odds of depression in women with early AAM was 1.27 (OR: 1.27, 95% CI: 0.96–1.69, *P* = 0.090), and 1.21 in women with normal AAM (OR: 1.21, 95% CI: 1.03–1.43, *P* = 0.024), compared to those with an AAM of 14 years and above (P for trend = 0.042).

Table 1 presents the frequency of depression and the results of univariate regression analysis for the odds of depression stratified by study variables.

**Table 1:** Frequency and crude OR of depression in TCS women according to demographic variables, BMI, PA and fertility

Variables		Total	Depression		Univariate logistic regression		
			n	%	OR	95% CI	P-value
Age	35–39	1026	97	9.5	Ref.	Ref.	Ref.
	40–49	2149	267	12.4	1.36	1.06–1.74	0.014
	50–59	1869	223	11.9	1.30	1.03–1.67	0.042
	60–70	1059	96	9.1	0.95	0.71–1.28	0.759
Residence	Urban	4066	601	14.8	4.13	3.26–5.24	< 0.001
	Mountainous	2037	82	4	Ref.	Ref.	Ref.
Socioeconomic level	1 (Lowest)	1395	75	5.4	Ref.	Ref.	Ref.
	2	1255	124	9.9	1.93	1.43–2.60	< 0.001
	3	1235	172	13.9	2.85	2.15–3.78	< 0.001
	4	1136	156	13.7	2.80	2.10–3.73	< 0.001
	5 (Highest)	1082	156	14.4	2.96	2.22–3.95	< 0.001
Education level	University/College	1041	113	10.9	Ref.	Ref.	Ref.
	9–12 years in school	1568	228	14.5	1.40	1.10–1.78	0.006
	6–8 years in school	618	95	15.4	1.49	1.11-2	0.007
	1–5 years in school	1657	185	11.2	1.03	0.80–1.32	0.803
	No schooling	1219	62	5.1	0.44	0.32–0.61	< 0.001
Occupation	No	4996	562	11.2	Ref.	Ref.	Ref.
	Yes	1107	121	10.9	0.97	0.79–1.19	0.761
Marital status	Single/widow	771	96	12.5	1.15	0.91–1.45	0.235
	marriage	5332	587	11	Ref.	Ref.	Ref.
AAM	≤ 11	528	67	12.7	1.27	0.96–1.69	0.090
	12–13	2379	289	12.1	1.21	1.03–1.43	0.024
	≥ 14	3196	327	10.2	Ref.	Ref.	Ref.
Menopause	No	3351	378	11.3	Ref.	Ref.	Ref.
	Yes	2752	305	11.1	0.98	0.83–1.15	0.808
BMI	< 25	1070	93	8.7	Ref.	Ref.	Ref.
	25-29.9	2447	254	10.4	1.22	0.95–1.56	0.123
	≥ 30	2586	336	13	1.57	1.23-2.00	< 0.001
MET (PA)	≥ Median	3120	443	14.2	Ref.	Ref.	Ref.
	< Median	2983	240	8	1.89	1.60–2.23	< 0.001
APG	Without pregnancy	364	28	7.7	Ref.	Ref.	Ref.
	< 20	2265	271	12	1.63	1.09–2.45	0.018
	20–24	2287	260	11.4	1.54	1.02–2.31	0.038
	25–29	842	97	11.5	1.56	1.01–2.42	0.047
	≥ 30	345	27	7.8	1.02	0.59–1.77	0.947
Number of pregnancy	0 or 1	733	66	9	Ref.	Ref.	Ref.
	2	1370	174	12.7	1.47	1.09–1.98	0.011
	3	1306	179	13.7	1.60	1.019–2.16	0.002
	4	925	99	10.7	1.21	0.87–1.68	0.252
	≥ 5	1769	165	9.3	1.04	0.77–1.40	0.799
OCP	No	3285	351	10.7	Ref.	Ref.	Ref.
	Yes	2818	332	11.8	1.12	0.95–1.31	0.176
Hysterectomy	No	5508	601	10.9	Ref.	Ref.	Ref.
	Yes	595	82	13.8	1.30	1.02–1.67	0.035

Table 2 illustrates the relationship between AAM and depression, adjusted for various covariates using multivariable logistic regression models. After adjusting for age, the odds of depression in women with early AAM was 1.27 (OR: 1.27, 95% CI: 0.96–1.68, *P* = 0.098), and 1.21 (OR: 1.21, 95% CI: 1.02–1.43, *P* = 0.027) for those with normal AAM, compared to those with late AAM (P for trend = 0.049). After adjusting for residential area, the odds of depression in women with early AAM was 1.03 (OR: 1.03, 95% CI: 0.78–1.37, *P* = 0.824), and 1.02 (OR: 1.02, 95% CI: 0.86–1.21, *P* = 0.796) for those with normal AAM, compared to those with late AAM (P for trend = 0.956)., After adjusting for BMI, the odds of depression in women with an early AAM was 1.21 (OR: 1.21, 95% CI: 0.91–1.61, *P* = 0.178), and 1.19 (OR: 1.19, 95% CI: 1.00-1.41, *P* = 0.045) in women with normal AAM, compared to those with late AAM (P for trend = 0.096). Similarly, after adjusting for hysterectomy, the odds of depression in women with an early AAM and normal AAM was 1.27 (OR: 1.27, 95% CI: 0.96–1.68, *P* = 0.092), and 1.21 (OR: 1.21, 95% CI: 1.02–1.43, *P* = 0.027), respectively, compared to women with late AAM (P for trend = 0.047). After adjusting for PA (measured by METs) the odds of depression in women with early AAM was 1.20 (OR: 1.20, 95% CI: 0.91–1.59, *P* = 0.202), and 1.15 (OR: 1.15, 95% CI: 0.97–1.37, *P* = 0.098) in those with normal AAM, compared to those with late AAM (P for trend = 0.178). Furthermore, after adjusting for the number of pregnancies, the odds of depression in women with early AAM was 1.24 (OR: 1.24, 95% CI: 0.94–1.65, *P* = 0.124), and 1.18 (OR: 1.18, 95% CI: 1.00-1.40, *P* = 0.048) in women with normal AAM, compared to those with late AAM (P for trend = 0.084). Also, after adjusting for age at first pregnancy (APG), the odds of depression in women with early AAM and normal AAM was 1.25 (OR: 1.25, 95% CI: 0.94–1.65, *P* = 0.124), and 1.18 (OR: 1.18, 95% CI: 1.00-1.40, *P* = 0.048), respectively, compared to those with late AAM (P for trend = 0.084).

**Table 2:** Crude and adjusted OR of the association between AAM and depression

Type of model	AAM	Logistic regression			P for trend
		OR	95% CI	P-value	
Crude model	≤ 11	1.27	0.96–1.69	0.090	0.042
	12–13	1.21	1.03–1.43	0.024	
	≥ 14	Ref.	Ref.	Ref.	
Adjusted age	≤ 11	1.27	0.96–1.68	0.098	0.049
	12–13	1.21	1.02–1.43	0.027	
	≥ 14	Ref.	Ref.	Ref.	
Adjusted area residence	≤ 11	1.03	0.78–1.37	0.824	0.956
	12–13	1.02	0.86–1.21	0.796	
	≥ 14	Ref.	Ref.	Ref.	
Adjusted BMI	≤ 11	1.21	0.91–1.61	0.178	0.096
	12–13	1.19	1.00-1.41	0.045	
	≥ 14	Ref.	Ref.	Ref.	
Adjusted hysterectomy	≤ 11	1.27	0.96–1.68	0.092	0.047
	12–13	1.21	1.02–1.43	0.027	
	≥ 14	Ref.	Ref.	Ref.	
Adjusted MET	≤ 11	1.20	0.91–1.59	0.202	0.178
	12–13	1.15	0.97–1.37	0.098	
	≥ 14	Ref.	Ref.	Ref.	
Adjusted by number of pregnancy	≤ 11	1.24	0.94–1.65	0.124	0.084
	12–13	1.18	1.00-1.40	0.048	
	≥ 14	Ref.	Ref.	Ref.	
Adjusted by APG	≤ 11	1.25	0.94–1.65	0.124	0.084
	12–13	1.18	1.00-1.40	0.048	
	≥ 14	Ref.	Ref.	Ref.	

Table 3 presents the results of multivariable logistic regression after adjustment for all confounders. The odds of depression in women with an early AAM and normal AAM was 0.97 (OR: 0.97, 95% CI: 0.73–1.29, *P* = 0.827) and 0.98 (OR: 0.98, 95% CI: 0.82–1.17, *P* = 0.830), respectively, compared to those with late AAM.

**Table 3:** OR of AAM related to depression in TCS based on multiple logistic regression model after adjustment for age, residential area, socioeconomic level, education, marital status, physical activity, hysterectomy, age at first pregnancy, BMI, number of pregnancies, and OCP use

Variables		Multiple logistic regression		
		OR	95% CI	P-value
Menarche age	≤ 11	0.97	0.73–1.29	0.827
	12–13	0.98	0.82–1.17	0.830
	≥ 14	Ref.	Ref.	Ref.

## Discussion

In the present study, the association between AAM and depression was examined in women from the TCS. The results indicated a significant trend in the odds of depression based on AAM. However, there was no significant association between AAM and depression after controlling for confounders. Our findings also highlighted the residential area (urban or mountainous) as a notable confounding factor with a substantial effect size, as the likelihood of depression remained nearly unchanged after controlling for residential area.

Regarding the relationship between AAM and depression, many studies suggested early AAM as a potential risk factor [22–24]. An epidemiological study in China on over 48 thousand females showed that a normal or late AAM (defined as ≥ 12 years old) was a protective factor against depression (OR: 0.94) [22]. Toffol et al. [23] studied 4391 female adults and found weak significant negative association between AAM and BDI questionnaire items. Their results also showed that AAM (as a continuous variable) is negatively associated with major depressive disorder (MDD) and major depressive episodes. Similarly, Harlow et al. [24], Mendle et al. [25], Askelund et al. [26], and Shen et al. [16] reported that the risk of depression increases with reductions in AAM with no observed differences among females with late and normal AAM. The difference between the results of these studies and the findings of the present study may be due to dissimilarities in the cultural background of the studied populations and variations in the size of studied subjects.

On a genetic view, the outcomes of a study on 630 females indicated a subtle difference where genetic inclinations towards late menarche were correlated with reduced depressive symptoms, while genetic inclinations towards early menarche were correlated with higher depressive symptoms. Nonetheless, this trend was discernible solely among girls hailing from more affluent socioeconomic contexts. Despite the observation that symptoms appeared to be independent of the timing of physical maturation in girls from economically disadvantaged backgrounds, these outcomes may still be influenced by genetic predisposition or environmental factors [27]. Similarly Wang et al. [28] found that individuals with a late genetically predicted AAM had lower risk of developing MDD and Sequeira et al. [29] demonstrated an association between early AAM and higher levels of depression using a genetic risk score. In contrast, Yu et al. [30] and Au Yeung et al. [31] found no effects from AAM on any psychiatric disorders after multivariable Mendelian randomization in the Chinese population. Additionally, on a disorder phenotypic view, Tondo et al. [32] found that earlier AAM was strongly associated with earlier age at onset of several psychiatric disorders such as bipolar disorder, MDD, and anxiety disorders.

There are also research that indicate late AAM as a potential risk factor for development of depression. Herva et al. [15] conducted a study on 3952 females with Finnish ethnicity. Their results showed that females with an AAM of 16 years and above are more prone to depression. This discrepancy may be due to differences between both AAM grouping and depression scales. Herva et al. [15] assessed for depression using Hopkins Symptom Checklist-25 (HSCL-25) and grouped AAM into three categories; 9 to 11, 12 to 15, and equal to 16 and beyond. Similarly, Kim et al. [33] also found that an AAM of ≥ 15 presents greater risk of depression compared to females with an AAM of ≤ 12 years in a large cohort of 945,729 Korean adults. However, similar to our findings, Opoliner et al. [34] studied 3711 females in the United States and found no association between either late or early AAM with depression.

Some studies were also conducted on non-general adult population samples to assess for associations between AAM and depression. Stumper et al. [35] assessed 140 adults in a psychiatry ward and Hirtz et al. [36] studied 184 girls in a psychiatry hospital. They both found that early AAM was associated with higher levels of depressive symptoms. Similarly, Joinson et al. [37] conducted a study on 2184 UK female children and found that girls with early menarche showed higher depression compare to normative and late menarche. However, it should be noted that Joinson et al. [37] used the Short Mood and Feelings Questionnaire (SMFQ) on girls with a mean age of thirteen years and 10 months. This should be considered when interpreting their results since the late AAM is mostly defined as an AAM of equal to and above fourteen years. Also, Bulhões et al. [38] studied 1988 thirteen year-old girls using the BDI-II questionnaire and considered pre-menarche girls as the reference population. They found that early AAM (defined as AAM ≤ 10) was associated with higher odds of depression (OR: 6.07) with a trend toward improvement with increase in AAM at 11, 12, and ≥ 13 (OR: 4.12, 3.59, and 2.89, respectively). In contrast, Zarate-Ortiz et al. [39] found no relationship between AAM and depression among girls with Mexican ethnicity.

A notable limitation when interpreting the findings of previous studies is the potential for selection bias. The majority of these studies have been conducted in high-income countries, focusing on populations from North America, Europe, and parts of Asia. This leaves regions such as Africa, the Middle East, and Latin America underrepresented, despite the likelihood that cultural and environmental factors in these areas may significantly influence both AAM and the risk of depression. Additionally, the variability in depression diagnostic methods across studies poses a challenge for comparing results. Differences in diagnostic criteria, assessment tools, and whether depression was clinically diagnosed by a physician, diagnosed based on questionnaires, or self-reported could contribute to inconsistencies in the findings. Expanding research to include more diverse geographic regions and standardizing diagnostic approaches would be essential for improving the generalizability and comparability of these studies.

## Limitations

The measurement of depression relied on documented self-reporting, which could potentially lead to underreporting, particularly as some participants may have been newly undiagnosed cases of depression. One limitation of this study is the lack of data on certain confounding variables in the initial cohorts registry that may influence both the AAM and the prevalence of depression. For instance, factors such as family history of mental health issues, exposure to environmental stressors, hormonal changes, childhood trauma, or early life adversities were not collected during the enrollment phase of the Tabari Cohort Study. While we adjusted for the confounding variables available in the dataset, the absence of these specific variables may limit our ability to fully account for their potential effects on the observed associations. Another key limitation of this study is its cross-sectional design, which prevents the establishment of causality. While our findings highlight an observed correlation between these variables, the directionality and underlying mechanisms remain unclear. Within the framework of the TCS, we intend to conduct longitudinal analyses following the completion of follow-up data collection that will allow us to assess causality and provide more comprehensive insights in the future studies. Also, cultural differences in nutrition, socioeconomic conditions, and health practices can affect the timing of menarche, while cultural norms and attitudes toward mental health may influence the expression and reporting of depressive symptoms. As the present study focuses on a population from Mazandaran Province in Iran, the findings may not be directly applicable to populations with different cultural backgrounds. Future studies should aim to replicate these findings in diverse cultural settings to enhance the generalizability of the results.

## Conclusion

Our results showed that although different AAM subgroups showed no relationship with depression on the multivariable model, but they showed a significant trend toward improvement in depression with higher AAM. Further research is needed to explore the underlying mechanisms between AAM and depression, including potential genetic and environmental influences.

## Electronic supplementary material

Below is the link to the electronic supplementary material.

Supplementary Material 1

## Data Availability

The data are available upon reasonable request from the corresponding author.

## Abbreviations

AAM: Age at menarche
OR: Odds ratio
CI: Confidence interval
TCS: Tabari cohort study
PERSIAN: Prospective Epidemiological Research Studies in Iran
PA: Physical activity
MET: Metabolic equivalent
BMI: Body mass index
OCP: Oral contraceptive
APG: Age at primigravida
MDD: Major depressive disorder
SMFQ: Short Mood and Feelings Questionnaire
