# Supplementary material for: The association between age at menarche and depression: a cross-sectional analysis of the TABARI cohort at enrollment phase
Source: BMC Psychiatry. 2025 Mar 25;25:277. doi: 10.1186/s12888-025-06667-w (PMC11938718; doi:10.1186/s12888-025-06667-w)
Supplement: Supplementary file 1 — Supplementary Material 1 [file 12888_2025_6667_MOESM1_ESM.docx]

**Supplementary Table 1.** An overview of the controversies in the results of the previous studies.

| **Study** | **Population** | **Diagnostic tool** | **Result** |
| --- | --- | --- | --- |
| Umeda et al. 2022 | 1171 Japanese females aged 20-75 | DSM-IV Interview | Higher odds of depression in early vs. normal AAM |
| Kim et al. 2021 | 367314 Korean females aged 12-18 | WHO Interview | Higher odds of depression in early vs. normal AAM |
| Shen et al. 2019 | 15674 US females aged ≥ 18 | PHQ-9 | Higher odds of depression in early vs. normal AAM  No significant difference between late vs. normal AAM  Higher odds of depression in late vs. early AAM |
| Sequeira et al. 2017 | 2208 English girls aged 18 | ICD-10 Interview | No significant difference between early vs. normal AAM  No significant difference between late vs. normal AAM  Higher odds of depression in late vs. early AAM |
| Opoliner et al. 2014 | 3711 US females aged 20-25 | CES-D | No significant difference between early vs. normal AAM  No significant difference between late vs. normal AAM  No significant difference between late vs. early AAM |
| Herva et al. 2004 | 3952 Finnish females aged 31 | HSCL-25 | No significant difference between early vs. normal AAM  No significant difference between late vs. normal AAM  No significant difference between late vs. early AAM |

CES-D: Center for Epidemiological Studies for Depression, HSCL-25: Hopkins Symptom Checklist-25, DSM-IV: Diagnostic and Statistical Manual of Mental Disorders IV, ICD-10: 10th revision of the International Statistical Classification of Diseases and Related Health Problems, PHQ-9: Patient Health Questionnaire, WHO: World Health Organization
